# Supplementary material for: Somites are a source of nephron progenitors in zebrafish
Source: Nat Commun. 2025 Jul 26;16:6914. doi: 10.1038/s41467-025-62259-y (PMC12297604; doi:10.1038/s41467-025-62259-y)
Supplement: Supplementary file 1 — Supplementary Information [file 41467_2025_62259_MOESM1_ESM.pdf]

Somites are a source of nephron progenitors in zebrafish

- Supplementary Information

Zhenzhen Peng[1], Thitinee Vanichapol[1], Phong Dang Nguyen[2,3], Hao-Han George Chang[1], Katrinka M. Kocha[4], Lori L. O'Brien[5], Peter D. Currie[2,6], Peng Huang[4], and Alan J. Davidson[1,*]

[1] Department of Molecular Medicine & Pathology, The University of Auckland, Auckland 1023, New Zealand.

[2] Australian Regenerative Medicine Institute, Monash University, Clayton, Victoria, Australia.

[3] Current address: Institut Curie, PSL University, Sorbonne Université, CNRS UMR3215, Inserm U934, Genetics and Developmental Biology, 75005 Paris, France.

[4] Department of Biochemistry and Molecular Biology, Cumming School of Medicine, Alberta Children's Hospital Research Institute, University of Calgary, Calgary, AB T2N 4N1, Canada.

[5] Department of Cell Biology and Physiology, University of North Carolina at Chapel Hill, Chapel Hill, NC27599, USA.

[6] EMBL Australia, Monash University, Clayton, Victoria VIC 3800, Australia.

**Correspondence: a.davidson@auckland.ac.nz*

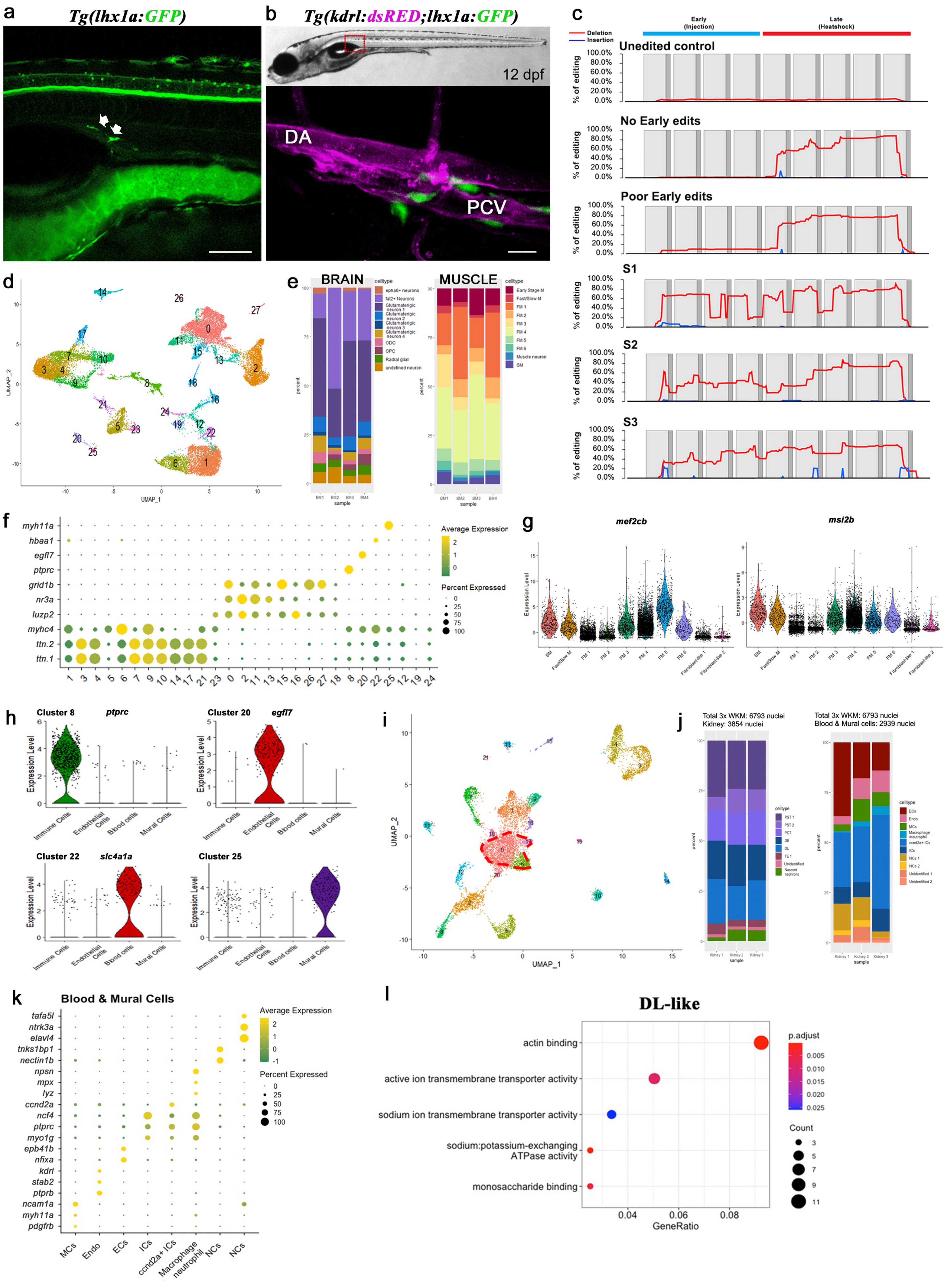
a
Tg(lhx1a:GFP)
b
Tg(kdrl:dsRED;lhx1a:GFP)
12 dpf
DA
PCV
c
Deletion
Insertion
Early
(Injection)
Late
(Heatshock)
Unedited control
No Early edits
Poor Early edits
S1
S2
S3
% of editing
100.0%
80.0%
60.0%
40.0%
20.0%
0.0%
d
UMAP_1
UMAP_2
e
BRAIN
MUSCLE
celltype
sample
percent
f
myh11a
hbaa1
egfl7
ptprc
grid1b
nr3a
luzp2
myhc4
ttn.2
ttn.1
Average Expression
Percent Expressed
g
mef2cb
msi2b
Expression Level
h
Cluster 8
ptprc
Cluster 20
egfl7
Cluster 22
slc4a1a
Cluster 25
Expression Level
Immune Cells
Endothelial Cells
Blood cells
Mural Cells
i
UMAP_1
UMAP_2
j
Total 3x WKM: 6793 nuclei
Kidney: 3854 nuclei
Total 3x WKM: 6793 nuclei
Blood & Mural cells: 2939 nuclei
k
Blood & Mural Cells
tafa5l
ntrk3a
elavl4
tnks1bp1
nectin1b
npsn
mpx
lyz
ccnd2a
ncf4
ptprc
myo1g
epb41b
nfixa
kdrl
stab2
ptprb
ncam1a
myh11a
pdgfrb
MCs
Endo
ECs
ICs
ccnd2a+ ICs
Macrophage neutrophil
NCs
NCs
Average Expression
Percent Expressed
l
DL-like
actin binding
active ion transmembrane transporter activity
sodium ion transmembrane transporter activity
sodium:potassium-exchanging ATPase activity
monosaccharide binding
GeneRatio
p.adjust
Count

Supplementary Fig. 1. Zebrafish brain, muscle and kidney single nuclei sequencing.
(a) Side view of a 12 dpf Tg(*lhx1a:GFP)* larvae showing GFP^+ NPCs around the axial vessels. Scale bar, 100µm. **(b)** Close-up of a 12 dpf Tg*(kdrl:dsRed;lhx1a:GFP)* larvae showing the GFP^+ NPCs in close proximity to the vasculature. Scale bar, 20µm. **(c)** GESTALT barcode editing efficiency. Editing rate detected in each of the nine CRISPR target sites of the GESTALT barcode plus control. Animals with high editing efficiency at all nine sites were selected for experiments (S1, S2 and S3). **(d)** UMAP showing the initial identification of 31 transcriptionally distinct muscle and brain clusters. **(e)** Percentage of nuclei per cluster from each of the 4 biological replicates of muscle and brain. **(f)** Dotplot showing key markers used to distinguish muscle and brain populations. **(g)** Violin plot showing the expression of *mef2cb* and *msi2b*, indicating the differentiating muscle clusters. **(h)** Violin plot showing the key marker for immune cells, endothelial cells, blood cells and mural cells from both muscle and brain tissue. Each cell type is color coded to match the highlights in UMAP. **(i)** UMAP showing the initial identification of 22 transcriptionally distinct kidney clusters. Cluster 0, 6 and 17 appeared to contain genes from multiple populations, suggesting poor quality that were possibly caused by the heat shock procedure during sample preparation (highlighted in red-dotted circle), thus removed for cluster identification. **(j)** Percentage of nuclei per cluster from each of the 3 biological replicates of kidney. **(k)** Dotplot showing key markers used to identify non-nephron populations. **(l)** Go-term analysis for the DL-like cluster.

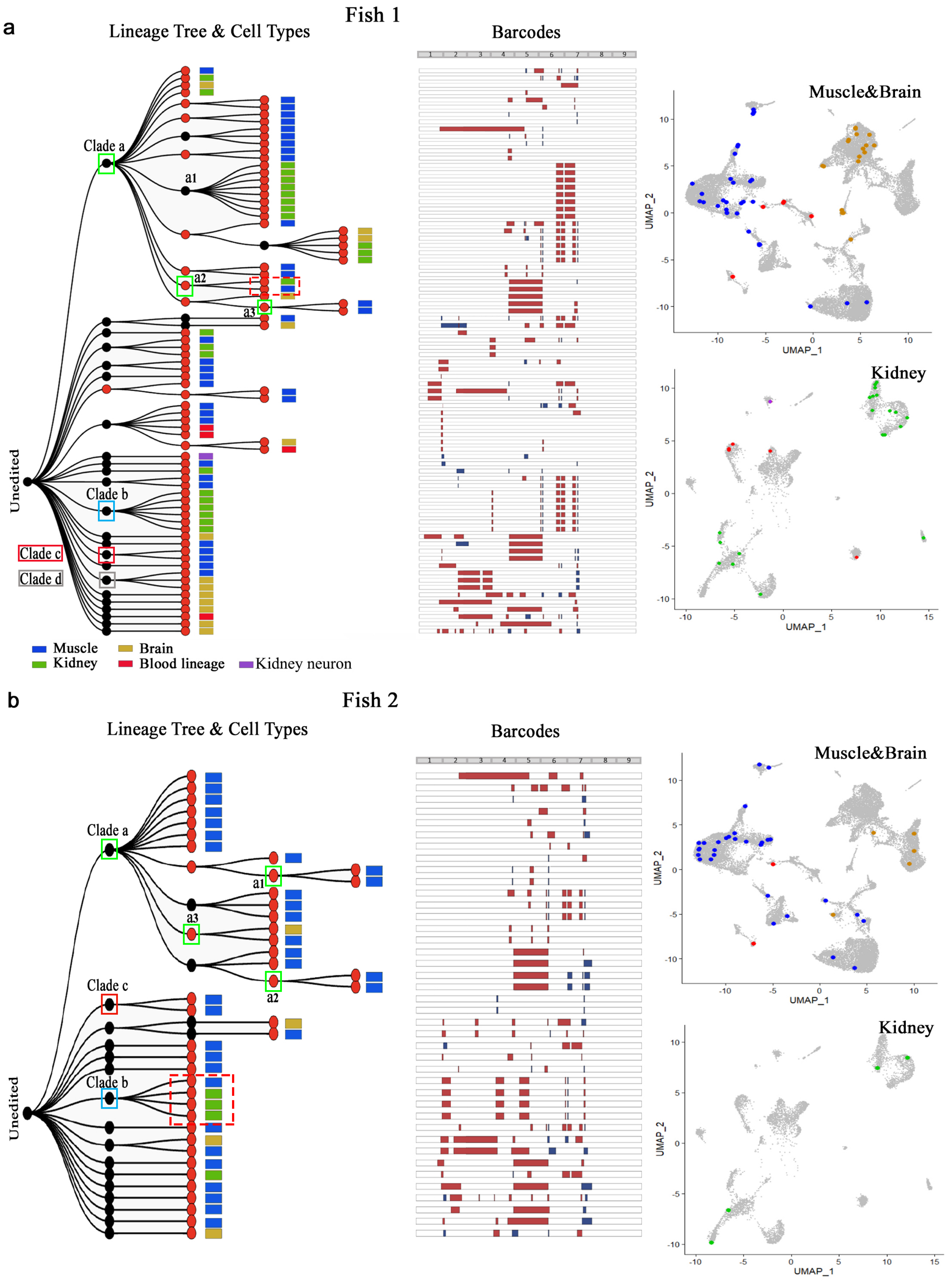
a
Fish 1
Lineage Tree & Cell Types
Barcodes
Muscle&Brain
Kidney
Clade a
a1
a2
a3
Clade b
Clade c
Clade d
Unedited
UMAP_1
UMAP_2
Muscle
Kidney
Brain
Blood lineage
Kidney neuron
b
Fish 2
Lineage Tree & Cell Types
Barcodes
Muscle&Brain
Kidney
Clade a
a1
a2
a3
Clade b
Clade c
Unedited
UMAP_1
UMAP_2

Supplementary Fig. 2. Reconstructed lineage tree of fish 1 and fish 2 kidney, muscle and brain using snGESTALT.

(a) Lineage tree of cells from Fish 1 based on barcode edits recovered from kidney, muscle, and brain tissues. Left: lineage tree constructed using shared barcode edits with a UMI threshold >2, based on a maximum parsimony approach. Each white bar displays the full barcode for a given cell, with deletions marked in red and insertions in blue. Black nodes represent edits at early barcode sites (1–4), while red nodes indicate edits at later sites (5–9). Black lines connect individual cells to shared nodes in the tree. Green-colored boxes denote clade 'a' and its subclades, with selected branches shown in Figure 2a. Right: UMAP plot showing the distribution of all cells from Fish 1 included in the lineage tree, with each dot representing a cell and color-coded by cell type. (b) Lineage tree of cells from Fish 2. Layout as in (a). Blue-, red-, and grey-colored boxes represent clades 'b', 'c', and 'd', respectively, with selected branches shown in Figure 2b. The accompanying UMAP plot shows the distribution of lineage-traced cells across kidney, muscle, and brain.

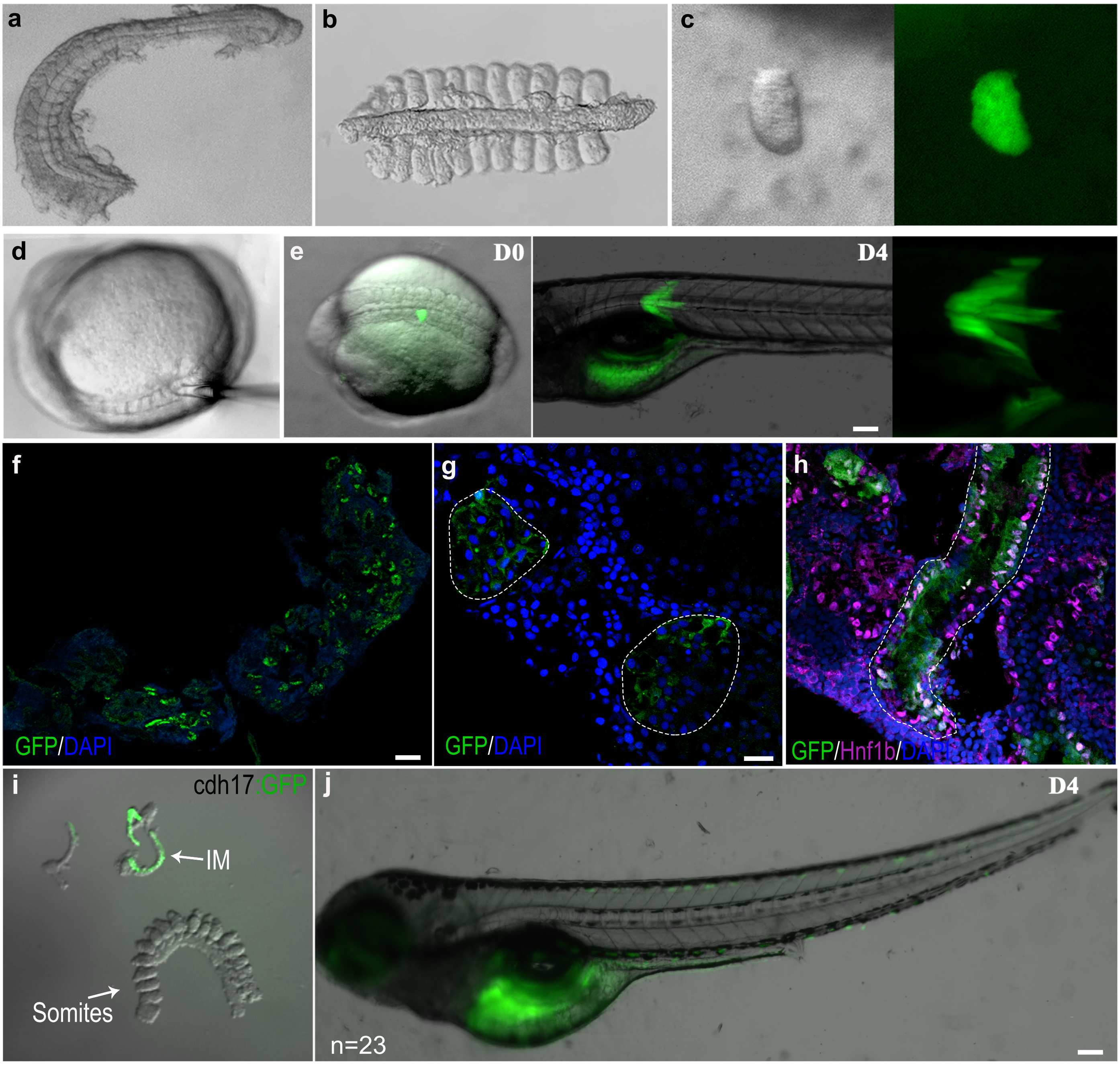

Supplementary Fig. 3. Overview of the somite transplantation procedure and formation of donor-derived nephrons from 18-somite stage.

(a-b) Collagenase digested donor embryo. **(c)** An isolated individual somite. **(d)** Image showing the removal of recipient embryo somite using a capillary needle. **(e)** Image showing recipient embryo carrying transplanted donor GFP^{+} somites, and the resulting GFP^{+} muscle fibers at 4 dpf. Scale bar, 100 µm. **(f-g)** Images of transplantation experiment at 18-somites stage, donor somite harvested at 18-somite stage and transplanted into an equivalent stage recipient embryo. **(f)** Low magnification image of a 1.5-month-old recipient fish with GFP^{+} donor derived tubules. Scale bar, 100 µm. **(g)** Donor derived GFP^{+} glomeruli in recipient kidney. Scale bar, 20 µm. **(h)** Donor derived GFP^{+}/Hnf1b^{+} tubules in recipient fish, n=2 independent biological replicates, Scale bar, 20 µm. **(i)** Collagenase-digested Tg(*cdh17*:GFP) 18-somite stage donor embryo, imaging showing complete and clean separation of IM (GFP^{+}) and somites (dark) after collagenase digestion. **(j)** 4 dpf recipient fish with transplanted somite from the Tg(*cdh17*:GFP) embryo showing no contribution to any GFP^{+} IM structure. n=23 independent biological replicates.

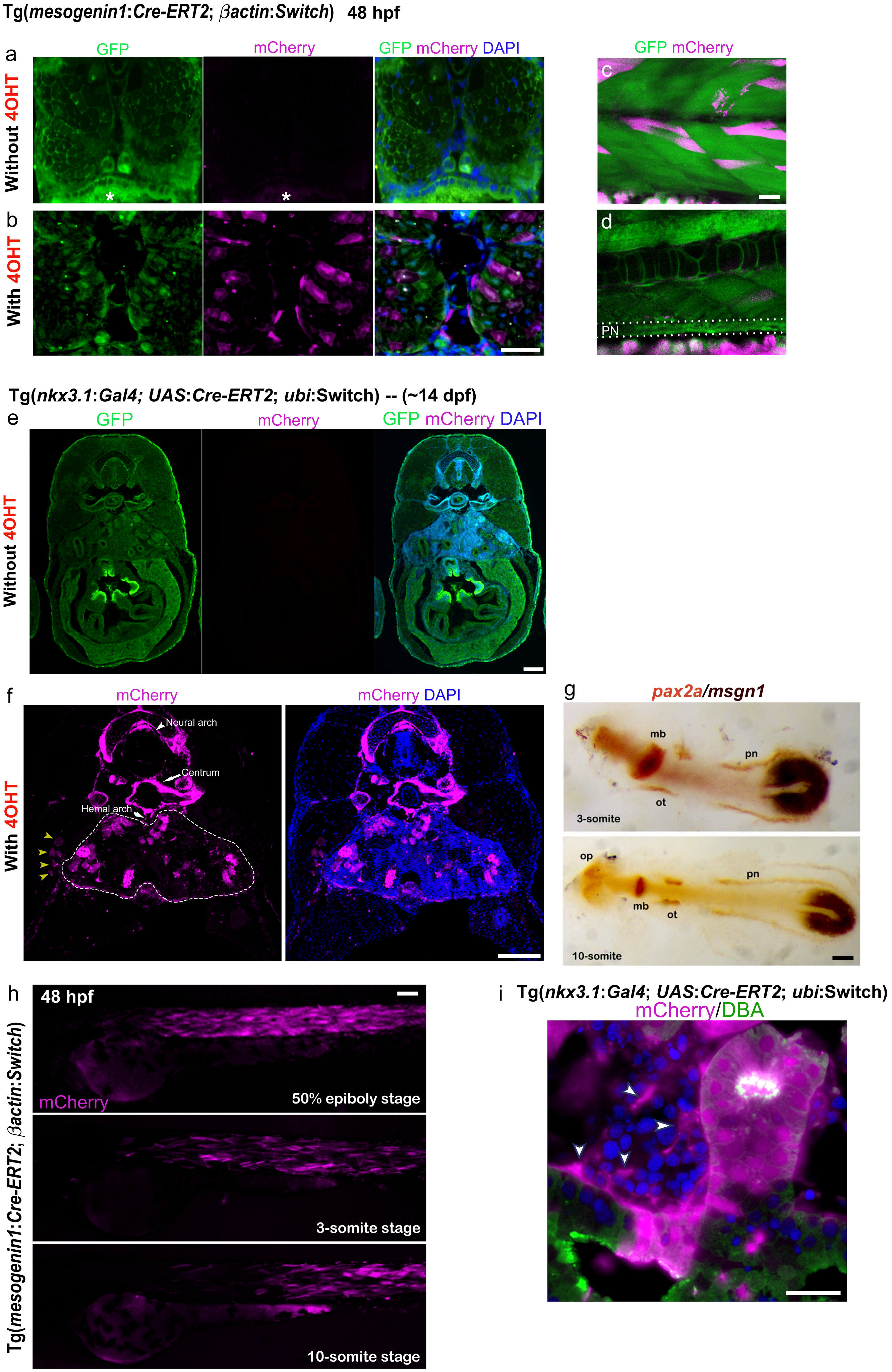

Tg(mesogenin1:Cre-ERT2; βactin:Switch) 48 hpf
a
b
GFP
mCherry
GFP mCherry DAPI
Without 4OHT
With 4OHT
*
*
GFP mCherry
c
d
PN
Tg(nkx3.1:Gal4; UAS:Cre-ERT2; ubi:Switch) -- (~14 dpf)
e
GFP
mCherry
GFP mCherry DAPI
Without 4OHT
f
mCherry
mCherry DAPI
With 4OHT
Neural arch
Centrum
Hemal arch
g
pax2a/msgn1
mb
pn
ot
3-somite
op
pn
mb
ot
10-somite
h
48 hpf
mCherry
50% epiboly stage
3-somite stage
10-somite stage
Tg(mesogenin1:Cre-ERT2; βactin:Switch)
i
Tg(nkx3.1:Gal4; UAS:Cre-ERT2; ubi:Switch)
mCherry/DBA

Supplementary Fig. 4. Expression of mCherry in both Tg(*msgn1*:Cre-ERT2;*bactin*:Switch) and Tg(*nkx3.1:Gal4; UAS:Cre-ERT2; ubi:Switch*) fish with and without 4-OHT.

(a-b) Cross-section of 48hpf *msgn1*-Cre larvae treated without (a) and with (b) 4-OHT. Scale bar, 100 µm. Images are representative of five biologically independent fish (n = 5), with similar results. **(c)** Representative confocal image of endogenous mCherry/GFP expression in muscle fibers of live, 4-OHT treated Tg(*msgn1*:Cre-ERT2;*bactin*:Switch) 7mm larvae, n=5 independent biological replicates. **(d)** Image of the same larvae in (c) showing lack of mCherry$^+$ cells in the pronephros (PN; dotted outline). Scale bar, 20 µm. **(e,f)** Cross-section of 7mm *nkx3.1-GAL-UAS-C*re larvae treated without **(e)** and with **(f)** 4-OHT. Scale bar, 100 µm. n = 5 independent biological replicates for each condition. **(g)** Double in situ of *pax2a* and *msgn1* of embryos at 3 and 10 somite stage, showing the relative location of intermediate mesoderm (IM, *pax2a*$^+$) and paraxial mesoderm (*msgn1*$^+$). Scale bar, 100 µm. n=3 fish embryos. (h) Expression pattern of *msgn1* in 48 hpf for embryos treated with 4OHT at three different time points: 50% epiboly, 3- and 10-somite stages Scale bar, 100 µm. Images are representative of ten biologically independent fish for each time points (n = 10), with similar results. (i) Cross-section of 1.5-month-old *nkx3.1-GAL-UAS-C*re fish kidney showing four distinct types of mCherry$^+$ kidney stromal cells (indicated by white arrows) located near the DBA-labelled distal tubule (green). Scale bar, 20 µm. Representative confocal image of three biologically independent fish, n=3.
