# Supplementary material for: Somites are a source of nephron progenitors in zebrafish
Source: Nat Commun. 2025 Jul 26;16:6914. doi: 10.1038/s41467-025-62259-y (PMC12297604; doi:10.1038/s41467-025-62259-y)
Supplement: Supplementary file 3 — Description of Additional Supplementary Files [file 41467_2025_62259_MOESM3_ESM.pdf]

Description of Additional Supplementary Files

File Name: Supplementary Data 1
Description: Differentially expressed genes (DEGs) for muscle clusters.

File Name: Supplementary Data 2
Description: DEGs for brain clusters.

File Name: Supplementary Data 3
Description: DEGs for kidney clusters.

File Name: Supplementary Data 4
Description: Top 20 genes for each kidney cluster.
