# Supplementary material for: Somites are a source of nephron progenitors in zebrafish
Source: Nat Commun. 2025 Jul 26;16:6914. doi: 10.1038/s41467-025-62259-y (PMC12297604; doi:10.1038/s41467-025-62259-y)
Supplement: Supplementary file 8 — Reporting Summary [file 41467_2025_62259_MOESM8_ESM.pdf]

nature portfolio

Corresponding author(s): Prof. Alan Davidson

Last updated by author(s): 09/07/2025

Reporting Summary

Nature Portfolio wishes to improve the reproducibility of the work that we publish. This form provides structure for consistency and transparency in reporting. For further information on Nature Portfolio policies, see our Editorial Policies and the Editorial Policy Checklist.

Statistics

For all statistical analyses, confirm that the following items are present in the figure legend, table legend, main text, or Methods section.

n/a	Confirmed	
☐	☒	The exact sample size (*n*) for each experimental group/condition, given as a discrete number and unit of measurement
☐	☒	A statement on whether measurements were taken from distinct samples or whether the same sample was measured repeatedly
☐	☒	The statistical test(s) used AND whether they are one- or two-sided<br>*Only common tests should be described solely by name; describe more complex techniques in the Methods section.*
☒	☐	A description of all covariates tested
☒	☐	A description of any assumptions or corrections, such as tests of normality and adjustment for multiple comparisons
☒	☐	A full description of the statistical parameters including central tendency (e.g. means) or other basic estimates (e.g. regression coefficient) AND variation (e.g. standard deviation) or associated estimates of uncertainty (e.g. confidence intervals)
☐	☒	For null hypothesis testing, the test statistic (e.g. *F*, *t*, *r*) with confidence intervals, effect sizes, degrees of freedom and *P* value noted<br>*Give P values as exact values whenever suitable.*
☒	☐	For Bayesian analysis, information on the choice of priors and Markov chain Monte Carlo settings
☒	☐	For hierarchical and complex designs, identification of the appropriate level for tests and full reporting of outcomes
☒	☐	Estimates of effect sizes (e.g. Cohen's *d*, Pearson's *r*), indicating how they were calculated

Our web collection on statistics for biologists contains articles on many of the points above.

Software and code

Policy information about availability of computer code

Data collection	Software used in this study for data analysis: Cellranger 6.0.1; R-studio 2023.09.1.
Data analysis	Sequencing data were demultiplexed and aligned to the zebrafish genome (GRCz10) using Cell Ranger v6.0.1 with the setting --include-introns.<br>All snRNA-seq data analyses were performed using standard protocols with previously described R packages: Seurat 4.0; SoupX 1.4.5; scDblFinder 1.4.0; ClusterProfiler 3.1.8 . GESTALT data were processed using Single-cell GESTALT pipeline available at GitHub (https://github.com/mckennalab/SingleCellLineage). The R scripts are available upon request.

For manuscripts utilizing custom algorithms or software that are central to the research but not yet described in published literature, software must be made available to editors and reviewers. We strongly encourage code deposition in a community repository (e.g. GitHub). See the Nature Portfolio guidelines for submitting code & software for further information.

Data

Policy information about availability of data

All manuscripts must include a data availability statement. This statement should provide the following information, where applicable:

- Accession codes, unique identifiers, or web links for publicly available datasets
- A description of any restrictions on data availability
- For clinical datasets or third party data, please ensure that the statement adheres to our policy

The snRNA-Seq data for this study have been deposited in the Gene Expression Omnibus under accession number GSE286280. The data is publicly available under https://www.ncbi.nlm.nih.gov/geo/query/acc.cgi?acc=GSE286280.

Research involving human participants, their data, or biological material

Policy information about studies with human participants or human data. See also policy information about sex, gender (identity/presentation), and sexual orientation and race, ethnicity and racism.

Reporting on sex and gender	*Use the terms sex (biological attribute) and gender (shaped by social and cultural circumstances) carefully in order to avoid confusing both terms. Indicate if findings apply to only one sex or gender; describe whether sex and gender were considered in study design; whether sex and/or gender was determined based on self-reporting or assigned and methods used. Provide in the source data disaggregated sex and gender data, where this information has been collected, and if consent has been obtained for sharing of individual-level data; provide overall numbers in this Reporting Summary. Please state if this information has not been collected. Report sex- and gender-based analyses where performed, justify reasons for lack of sex- and gender-based analysis.*
Reporting on race, ethnicity, or other socially relevant groupings	*Please specify the socially constructed or socially relevant categorization variable(s) used in your manuscript and explain why they were used. Please note that such variables should not be used as proxies for other socially constructed/relevant variables (for example, race or ethnicity should not be used as a proxy for socioeconomic status). Provide clear definitions of the relevant terms used, how they were provided (by the participants/respondents, the researchers, or third parties), and the method(s) used to classify people into the different categories (e.g. self-report, census or administrative data, social media data, etc.) Please provide details about how you controlled for confounding variables in your analyses.*
Population characteristics	*Describe the covariate-relevant population characteristics of the human research participants (e.g. age, genotypic information, past and current diagnosis and treatment categories). If you filled out the behavioural & social sciences study design questions and have nothing to add here, write "See above."*
Recruitment	*Describe how participants were recruited. Outline any potential self-selection bias or other biases that may be present and how these are likely to impact results.*
Ethics oversight	*Identify the organization(s) that approved the study protocol.*

Note that full information on the approval of the study protocol must also be provided in the manuscript.

Field-specific reporting

Please select the one below that is the best fit for your research. If you are not sure, read the appropriate sections before making your selection.

[x] Life sciences   [ ] Behavioural & social sciences   [ ] Ecological, evolutionary & environmental sciences

For a reference copy of the document with all sections, see nature.com/documents/nr-reporting-summary-flat.pdf

Life sciences study design

All studies must disclose on these points even when the disclosure is negative.

Sample size	Biological replicate numbers per condition varied between n=3 and n=10, depending on the specific assay and experimental feasibility. These sample sizes are consistent with established norms for this type of assay. While no formal power analysis was conducted, results were reproducible across independent experiments.
Data exclusions	No data were excluded from the analysis.
Replication	To verify reporoducibility, all key biological experiments were performed in triplicate, and consistent results were observed across independent replicates. For somite transplant experiments, over 100 transplants were performed, of which five yielded successful donor integration. These successful cases showed consistent phenotypic outcomes, supporting the robustness of the findings despite the technical challenges and low efficientcy of the procedure. For the Gestalt analysis, the experiment was performed once due to cost constraints. However, high statistical power was achieved by analyzing over 33,000 individual nuclei, allowing robust detection of patterns across a large sample population. The results were internally consistent and supported the conclusions drawn.

Randomization	samples allocated randomally
Blinding	Experiments were not blinded, as blinding is not standard practice for this type of assay. Data interpretation was guided by predefined criteria to reduce potential bias.

Reporting for specific materials, systems and methods

We require information from authors about some types of materials, experimental systems and methods used in many studies. Here, indicate whether each material, system or method listed is relevant to your study. If you are not sure if a list item applies to your research, read the appropriate section before selecting a response.

Materials & experimental systems

n/a	Involved in the study	
☐	☒	Antibodies
☒	☐	Eukaryotic cell lines
☒	☐	Palaeontology and archaeology
☐	☒	Animals and other organisms
☒	☐	Clinical data
☒	☐	Dual use research of concern
☒	☐	Plants

Methods

n/a	Involved in the study	
☒	☐	ChIP-seq
☒	☐	Flow cytometry
☒	☐	MRI-based neuroimaging

Antibodies

Antibodies used	anti Pax2 (1:500, Covance, PRB-276P-200), anti Hnf1b (1:500, Sigma, HPA002083-100UL), anti GFP (1:500, Novus, NOVNB600597), anti mCherry (1:500, Abcam, ab125096), anti Podxl (1:250, made in House by Dr. Hidetake Hurihara, Juntendo University). Donkey anti-mouse/Rabbit IgG (H+L) Highly cross-adsorbed Secondary antibodies (1:500, Thermo Fisher Scientific)
Validation	Anti-Pax2 (Covance, PRB-276P-200, RRID:AB_291611): Species reactivity: Mouse, Rat, Human, zebrafish. Application(s): IF, IHC. This antibody is reported by the manufacturer to be validated for use in IHC and IF across multiple species. It has been widely used in peer-reviewed publications. (Li X, et al. 2023. Nat Neurosci).<br><br>Anti-Hnf1b (Sigma, HPA002083): Species reactivity: Mouse, Human, zebrafish. Application(s): IF, IHC. Used in zebrafish studies to mark renal progenitors. This antibody is part of the Human Protein Atlas project and has been validated for IHC and IF in human and mouse tissues. Although zebrafish is not officially listed by the manufacturer under species reactivity, this antibody has been successfully used in peer-reviewed zebrafish studies (e.g., Naylor et al., 2013; PMID: 23559574), supporting its cross-species utility.<br><br>Anti-GFP (Novus, NB600-597) Widely used and validated antibody for detecting GFP across multiple species, including zebrafish. Recognizes native and transgenic GFP expression. This antibody has been widely cited in peer-reviewed literature (e.g. Wolf et al., 2020).<br><br>Anti-mCherry (Abcam, ab125096) Species reactivity: Reacts with mCherry fluorescent protein (used in transgenic models across species). The manufacturer reports validation for IF, IHC, and WB. This antibody has been cited in over 180 peer-reviewed publications. Also, commonly used in zebrafish to detect mCherry fusion proteins and lineage reporters (e.g., Ye et al., Nature Comm 2023).<br><br>Anti-Podxl (Podocalyxin) – custom antibody generated and validated by Dr. Hidetaka Hirahara (Juntendo University). Antibody shows expected podocyte-specific staining pattern in zebrafish pronephros consistent with published expression domains.<br><br>Secondary antibodies: Donkey anti-mouse and anti-rabbit IgG (H+L), Alexa Fluor-conjugated (Thermo Fisher Scientific) Highly cross-adsorbed secondaries validated by manufacturer for immunofluorescence to minimize cross-reactivity.

Animals and other research organisms

Policy information about studies involving animals; ARRIVE guidelines recommended for reporting animal research, and Sex and Gender in Research

Laboratory animals	Zebrafish (Danio rerio) of mixed sexes, under 30 days post-fertilization (dpf) or 1.5 months of age, were used.<br>Tg(lhx1a:EGFP) from Prof Hukriede's lab, University of Pittsburgh, background: AB<br>Tg(hsp70l:Cas9-t2A-GFP, 5xU6:sgRNA): generated in our lab using Addgene #108871, background: AB<br>Tg(hspDRv7:GESTALT, clmc2:EGFP): generate in our lab using Addgene #108870, background: AB<br>Tg(bactin2:switch) from Australian Regenerative Medicine Institute, Monash University, Clayton, background: AB<br>Tg(msgn1:Cre-ERT2) from Australian Regenerative Medicine Institute, Monash University, Clayton, background: AB<br>TgBAC(nkx3.1:Gal4)ca101, Tg(UAS:Cre-ERT2)ca105 and Tg(Ubi:Switch): all three lines were maintained in Associate Professor Huang's lab (Cumming School of Medicine, University of Calgary). Background: AB

Wild animals	The study did not involve wild animals
Reporting on sex	Sex of the fish is not considered in the study
Field-collected samples	The study did not involve samples collected from field
Ethics oversight	This study was approved by the University of Auckland Animal Ethics Committee under approved protocol AEC22634.

Note that full information on the approval of the study protocol must also be provided in the manuscript.

Plants

Seed stocks	*Report on the source of all seed stocks or other plant material used. If applicable, state the seed stock centre and catalogue number. If plant specimens were collected from the field, describe the collection location, date and sampling procedures.*
Novel plant genotypes	*Describe the methods by which all novel plant genotypes were produced. This includes those generated by transgenic approaches, gene editing, chemical/radiation-based mutagenesis and hybridization. For transgenic lines, describe the transformation method, the number of independent lines analyzed and the generation upon which experiments were performed. For gene-edited lines, describe the editor used, the endogenous sequence targeted for editing, the targeting guide RNA sequence (if applicable) and how the editor was applied.*
Authentication	*Describe any authentication procedures for each seed stock used or novel genotype generated. Describe any experiments used to assess the effect of a mutation and, where applicable, how potential secondary effects (e.g. second site T-DNA insertions, mosiacism, off-target gene editing) were examined.*
